# Effect of Different Forms of Silver on Biological Objects

**DOI:** 10.1134/S2635167622020021

**Published:** 2022-05-21

**Authors:** A. A. Antsiferova, P. K. Kashkarov, M. V. Koval’chuk

**Affiliations:** 1 National Research Center “Kurchatov Institute”, Moscow, Russia; 2 Moscow Institute of Physics and Technology, Moscow, Russia; 3 Moscow State University, Moscow, Russia

## Abstract

Silver has been known since ancient times on account of its pronounced antiseptic properties. Currently, its antibacterial, antiviral, and fungicidal properties are highly desired in the food and cosmetic industries, in medicine, and pharmacology. Silver exhibits toxic effects not only on pathogenic organisms but also on healthy cells. Over the past 20 years, nanosilver, a new form of silver, has been introduced in various areas of industry. The transition to the nanoscale form results in the revision of standard approaches to items, including those based on this element, and the emergence of such a novel research area as nanosafety. In this review, we address the history of using different forms of silver, the mechanisms of its interaction with living cells, toxic properties, biokinetic parameters, capability for accumulation in different organs, effects on cognitive functions, and the clinically known argyrosis condition. Relevant publications are critically analyzed and conclusions are drawn. The broader incorporation of such a weakly biophilic element as silver in the biosphere and ecosphere calls for our understanding of biochemical processes underlying the interaction of this element, in its different forms, with living cells and multicellular organisms.

## CONTENTS

Introduction

1. Uses of silver

2. Mechanisms of interaction between different forms of silver and biological organisms

3. Toxicity of silver compounds

4. Biokinetics of silver

5. Effect of silver on cognitive functions in mammals

6. Argyrosis

Conclusions

## INTRODUCTION

The levels of silver in a human body living in the natural environment and technosphere are detectable by modern analytical techniques [1], and fairly specific average values were reported in some published sources. Thus, the expected level of Ag in the human body and in animals was reported to be 20 μg per 100 g on a dry matter basis [2]. The metal is stored predominantly in brain cells, endocrine glands, the liver, and the kidneys.

Silver is not a biophilic element for humans, animals, and plants, and it is not involved in critical biochemical processes [3]. As a result of the development of medicine, cosmetology, and the food industry, the use of silver, and therefore the exposure of potential consumers as well as workers directly involved in its production, is beyond doubt. In fact, the metal is fairly stable due to its relatively low chemical reactivity, which may be critical in its uncontrolled reclamation. These are the causes of the accumulation of silver in the ecosphere and representatives of the biosphere, with subsequent inclusion in trophic chains. As a result, due to low biophilicity and, probably, low biocompatibility of silver, there are potential risks associated with the active use of this element in various processes in the technosphere.

In addition, the diversity of different forms of chemical compounds has broadened. The development of nanotechnology has enabled the syntheses of various chemicals, including nanoscale forms of silver. The current thought is that the transition to the nanoscale, which imparts new (including biological-like) properties to synthetic materials [4], requires us to develop separate picture of their interaction with living beings [5].

Taking into account interactions between silver and living beings, the transition to nanoscale forms brings even greater uncertainty. All this substantiates the importance of developing careful hygiene and toxicology characteristics of silver compounds taking into account their form. Conclusions about potential risks associated with increasing use of this element can be framed by analyzing the results of a sizable number of in vitro, in vivo, and *in silico* experiments.

To obtain a preliminary objective estimate of the human intake of silver via natural routes, we should refer to current norms. Thus, according to WHO reports from 2008, the average human daily intake of Ag is 7 μg [6]. At the same time, the U.S. National Institute for Occupational Safety and Health specifies the upper limit for all forms of silver to be 0.01 mg/m^3^ [7]. Due to the fact that use of silver in industry has grown over recent years, it is important to highlight that there is a high probability that the content of silver in drinking water will rise.

The present review concentrates on the problems of interaction between silver compounds in different forms and biological organisms. We consider several areas of their current application; possible mechanisms of interactions with cells; and concurrent effects such as toxicity, accumulation in the body, and changes in cognitive functions. Argyrosis, a condition affecting subjects who consume silver over prolong periods, is considered in a separate section. Conclusions on the potential risks associated with the uses of silver in the modern world will be presented.

## 1 USES OF SILVER

Normally, silver is associated with jewelry. Its high inertness and stability are key properties associated with the traditional use of silver and its alloys in this industry. The disinfecting properties of silver have been known since ancient times. For instance, silver items were used for medical purposes in ancient Egypt and Mesopotamia. Hindu (Ayurvedic and religious) literature mentions a way of disinfecting water by immersing white-hot silver in it or upon prolonged contact with metallic silver under regular conditions [8]. Similarly, the effective use of the properties of silver by Knights Hospitaller in the XVI century during an unsuccessful attempt to capture the island of Malta by Ottoman Turks is mentioned [9]. The Hospitallers paid special attention to hygiene: they preferred large airy spaces and silver kitchenware, whereas the Ottoman army used wooden dishes. The Knights deliberately poisoned water in wells along routes of approach with the corpses of animals and Ottoman warriors. Intestinal infections began to spread among the Ottomans as a result, which noticeably weakened their military capacity and ultimately the Turks had to withdraw and leave the island.

Up until the 1800s, silver had been used exclusively in its metallic form [10]. On account of Jenner’s studies on vaccination and Paster, Koch, and Ehrlich’s studies on the pathogenicity of infectious diseases, the aseptic nature of silver had become clearer by 1930, and the diversity of its forms had also broadened. Thus, colloidal silver (Argyrol, Protargol), silver nitrate, and silver−arsenic compounds began to be used. The period from 1930s to 1970s was marked by the emergence of modern antimicrobial chemotherapy and the discoveries of sulfanilamide and penicillin. As a result of the emergence of antibiotics, the medicinal use of silver progressively declined. In the 1960s, however, when Monafo and Moyer began to use 0.5% silver-nitrate solutions in burn-wound care, silver compounds were revived [11]. In 1968, Fox introduced 1%-silver-sulfadiazine cream in medical practice [12], and up to the present day it has remained one of the most commonly used external medicines for the treatment of burns.

New pharmaceuticals based on silver compounds found widespread use beginning in the 1970s due to the development of novel methods for the identification, culturing, and typification of bacteria; approaches to antimicrobial susceptibility testing; and technologies of wound-dressing manufacture. Silver-coated catheters and surgical needles began to be commonly used. Silver−protein compounds (Argyrol, Protergol, and Collargol) and silver−polymer compounds (Argovit) came into widespread use. The indicated compounds were more stable and more bioavailable due to the presence of a stabilizing capping layer. Essentially, these were Ag nanoparticles (NPs) [9]. Pharmaceuticals of this type are recommended for use in cosmetology (Agrokrem) for the treatment of inflammatory skin conditions, in purulent surgery, and in dentistry for the treatment of stomatitis and gingivitis (Argogel) [13]. It is important to mention the advantageous use of wound dressings containing ultradispersed nanocrystalline silver [14]. The good disinfecting and wound-healing properties of such products have been supported by extensive research [15, 16]. Similarly, nanosilver is fairly commonly used as a food supplement, especially outside of Russia. A series of silver compounds are used in antibacterial soaps, toothpastes, face creams, washing powders, and as preservatives in packing materials to extend the shelf-life of food products [10, 17, 18]. Nanosilver is also used in textiles for the treatment of fungal diseases and protection against viral infections. Thus, the problem of boosting the effectiveness and service life of textile materials (face masks) with Ag NPs is intensively discussed regarding identification of the optimal production technology for them [19]. As a result of the pandemic of the new coronavirus infection SARS-CoV-2, there is the demand for disinfecting agents and hand sanitizers based on Ag NPs due to their pronounced antiviral properties [20]. In addition, a COVID-19 vaccine containing spherical NPs as the carrier has been patented [21]. It is known that many synthesis technologies can be used for the relatively inexpensive commercial production of spherical Ag NPs. It can be expected that due to their low reactivity Ag NPs will find use in vaccine production.

As a result, in consideration of the demand for production and use of various silver compounds, it is obvious that the environment is becoming increasingly contaminated with this, strictly speaking, non-biophilic element, which may present new risks for representatives of the biosphere, humans, and for the environment as a whole. Under such circumstances, it is important to study possible concurrent effects that manifest during the interaction between silver and living objects and to understand the underlying mechanisms.

## 2 MECHANISMS OF INTERACTION BETWEEN DIFFERENT FORMS OF SILVER AND BIOLOGICAL ORGANISMS

The pronounced antibacterial, antiviral, and fungicidal properties of various silver compounds are well-documented [9, 22, 23]. These properties have ensured persistent interest in Ag-based medicines on the part of both medical professionals and researchers. The emergence of antibiotic-resistant strains has spurred intensive search for optimal (in terms of benefits vs. side effects) forms of silver [24]. Reports of a synergistic effect due to the concurrent use of silver compounds and antibiotics are not uncommon [25]. Thus, multiple enhancement of the antimicrobial activity was observed when silver ions were co-administered with ampicillin (tenfold), ofloxacin (tenfold), norfloxacin (tenfold), gentamycine (hundredfold), tobramycin (threefold), and vancomycin (tenfold). Similarly, reductions in the minimal inhibitory concentration were observed with tobramycin (tenfold), polymyxin B (by 5−10 times), and tetracycline (twofold) [26]. A synergistic effect was observed when combining Ag NPs and antibiotics (ampicillin, streptomycin, rifampicin, and tetracycline) [22]. There is evidence, however, that such effects were absent for combinations of Ag NPs and ceftazidime, oxacillin, ciprofloxacin, and meropenem [27].

Lack of enhancement despite the presence of NPs was attributed to the growth of biofilms, which are the absolutely dominant form of existence of microorganisms in the human body affected by an infectious disease. To gain a better understanding of this phenomenon, we consider the mechanism of interaction between ionic forms of silver and cells, taking bacterial cells as an example. Silver cations can be produced upon dissociation in solutions of silver salts such as AgNO_3_ (silver nitrate), AgC_2_H_3_O_2_ (silver acetate), Ag_3_C_6_H_5_O_7_ (silver citrate), [Ag(NH_3_)_2_]ОН (silver diamine hydroxide), and some others.

Silver is thought to attack various macromolecules in bacteria. The following alterations of macromolecules were identified in bacteria exposed to silver: DNA condensation, membrane and protein damage, interactions with thiol groups, destabilization of iron-sulfide compounds, disruption of iron metabolism and homeostasis, and the substitution for metals in metalloproteins [5]. The microscopic analysis of Ag-treated bacteria showed the presence of a region of higher electronic density, which was condensed DNA in the center of cells. In vitro studies provided support for the hypothesis that silver may contribute to DNA modification thus creating a basis for mutations and replication inhibition [28]. These changes result in the generation of reactive oxygen species (ROS) and an increase in membrane permeability in Gram-negative bacteria, which may potentiate the activity of a large variety of antibiotics against Gram-negative bacteria at different metabolic states, as well as to make resistant bacterial strains sensitive to antibiotics again [29, 30].

It is believed that anions may contribute to the antiseptic effect, along with silver cations, which has been substantiated by observations of the comparable antibacterial activity of nitrates of other metals, such as tin, sodium, zinc, and cobalt [23].

Most generally, the effects of silver salts tend to be attributed to electrostatic interaction and the formation of chemical bonds between the respective ions and biomacromolecules in the cell. Similarly, they can form bonds to proteins and peptidoglycans in the plasma membrane [5]. Penetration into the intracellular space may occur by passive diffusion or, e.g., micropinocytosis.

A considerable tendency to form compounds with such biophilic elements as sulfur and selenium has been noted. These interactions are attributed to the high affinity between Ag and S through binding with thiol groups. The resulting Ag_2_S is highly stable and poorly soluble in water. It is suspected that selenium subsequently substitutes sulfur to form Ag_2_Se, which is an even more stable and less water-soluble compound [30]. Similarly, silver directly binds with Se within the enzyme glutathione peroxidase to form stable and chemically inert Ag_2_Se [31]. The properties listed above are important when considering the clinical consequences of the human use of silver-based pharmaceuticals, which will be addressed later in the text.

For a multicellular organism, the mode of action of silver ions may consist in the incorporation of silver ions into signaling pathways and transduction processes, resulting in their modification.

We note that cells have some specific defense mechanisms to counteract the effects of heavy metals. In bacteria, such mechanisms can be divided into endogenous and exogenous. The endogenous mechanisms involve mutations resulting in disappearance of the OmpC/F membrane protein responsible for the transport of silver ions inside the cell. The *E. coli* strain BW25113 with resistance to silver developed after 6-day exposure to subinhibitory concentrations of this metal [32]. The exogenous mechanism involves special cell-membrane proteins, which are responsible for the outflow of silver ions from the cell [30]. The presence of such ions triggers genetic alterations that enhance the outflow of the initiator.

For instance, in 1975, a strain of *Salmonella typhimurium* caused the death of several patients in the Burn Unit of Massachusetts General Hospital. The isolated pathogen proved to be resistant to silver on account of the presence of pMG101 plasmid [33]. pMG101 plasmid controls the resistance of bacteria to Hg and Ag metals, telluride, and such antibiotics as streptomycin, tetracycline, and ampicillin [34].

The listed mechanisms were observed in Gram-negative bacteria. Apparently, Gram-positive bacteria cannot develop resistance to silver.

It would not be a surprise if similar defense mechanisms are revealed in some types of cells in a more evolved organism. The competition of silver with other microelements, e.g., copper, can be an indirect consequence of these mechanisms [35].

The biological activity of bulk silver is also due to the partial dissociation into ions, which occurs relatively inactively due to its chemical inertness. Nevertheless, silver was found to exhibit an oligodynamic behavior, i.e., it is effective at low concentrations [36].

It is obvious that the biological activity of silver compounds is controlled by their solubility. It is proposed to discern the maximum allowable concentrations of silver in water in consideration of the solubility of corresponding compounds. Thus, the maximum allowable concentration for metallic silver is 0.1 mg/m^3^, whereas the value is 0.01 mg/m^3^ for its soluble forms [7].

The biological activity and bioavailability of Ag NPs is a separate problem. The current thought is that the transition to the nanoscale improves the bioavailability of a substance [37, 38]. The high surface-to-volume ratio of nanoscale particles is high, which means that a large number of reactive surface atoms is available and, therefore, there is a high chemical reactivity. Similarly, nanoscale dimensions ensure a high penetrating ability. In this context, NPs are able to pass through blood-tissue barriers and penetrate inside cells by both simple diffusion and various endocytosis pathways. This ability of NPs is apparently due to to their property to mimic cellular peptides, which, in their native state, fall in the nanoscale size range. As a result, the cell erroneously takes the NPs for a building material, signal molecules, or other vital compounds and subsequently internalizes them [39]. In doing so, the NPs can acquire a protein coating, which oftentimes is a determinant [40].

Recent technologies enable the synthesis of NPs with different shapes, sizes, and different types of stabilizing capping layers. It is the capping layer that controls the solubility of NPs and prevents them from aggregating. In some cases, the use of colloids of Ag NP without a stabilizing capping layer is complicated. Solutions of this type where the particle concentration is larger than 100 mg/L are unstable and prone to aggregation and sedimentation [23].

In addition to that, the behavior of stabilized NPs in different compartments of a biological organism, which have different pH, is unpredictable and questionable in many cases. The capping layer, on the one hand, can improve the bioavailability of NPs and facilitate their attachment to plasma membranes via weak physical interactions. But it can prevent the release of ions and hinder chemical reactions between the NPs and biomacromolecules, on the other. The latter point also accounts for the inactivation of Ag NPs in the case of biofilm formation [22].

The mechanism of interaction between NPs and the cell (e.g., the bacterial cell) has not been fully elucidated yet. It is suspected that Ag NPs can be a source of Ag^+^ ions in both extracellular and intracellular spaces. The effects of silver ions, which were considered above, consist in the interaction with biomacromolecules, DNA, and RNA; they boost ROS generation, cause genetic alterations, interfere with metabolism, and result in apoptosis and necrosis [5, 35, 41]. There is also the possibility that zero-valent silver may interact with biomacromolecules in the cell, with similar implications. Most likely the NPs will partly dissociate into ions, and both mechanisms may operate in parallel. For a multicellular organism, the ability of NPs to cause an immune response and inflammation is investigated [42].

Apparently, the key qualitative difference between NPs and ions, in terms of their effects on biological objects, is the long-term action of the former. Under certain conditions, NPs can be transported essentially unchanged to different compartments of the organism, where they will be stored and function as ion stores. Such possibilities were suggested by their short-term presence in blood and accumulation in internal organs [39, 43]. This behavior, in particular, can be specific to Ag NPs.

Whilst several questions concerning the mechanisms of interaction between silver compounds and living systems still remain, it is clear that their ability to form solutions is crucial. The current thought is that their biological activity is determined specifically by this property.

## 3 TOXICITY OF SILVER COMPOUNDS

Despite the advantages of the antimicrobial effects of silver, which were described above in detail, it is obvious that similar effects apply to nonpathogenic cells. This phenomenon is responsible for the potential toxicity of silver compounds and is particularly important when considering the exposure of multicellular organisms to this element. Admittedly, as Paracelsus said, “Solely the dose determines that a thing is not a poison.” [44] This was substantiated in numerous in vitro and in vivo scientific studies [45–48].

In several cases, in studying the effects of different doses of silver, dose- and time-dependent effects were observed. It was noted that the ionized form was more toxic than Ag NPs and, especially, bulk silver [45].

The toxicity of silver was observed in in vitro studies concerned with its interaction with a large variety of cell cultures. Its biological activity was found to exhibit common features. Thus, an increase in the ROS production, in particular, the superoxide radical, was observed. A decrease in the cell’s antioxidant defense system was also noted along with oxidative stress.

Of course, each cell line has its own unique features associated with the functions and structure of corresponding cells. Under such circumstances and in view of the great diversity of silver compounds (in particular, their nanoscale forms) [42], predicting the outcome can be quite complicated. As a result of the fact that biological organisms consist of a large number of different types of interrelated cells, results of in vivo studies vary and depend on many factors.

We note that in vitro studies are used more commonly because they are more time-efficient and economic. It was proposed to combine the results of in vivo experiments on toxicity with *in silico* results on biodistribution in order to predict in vivo toxicity for each particular tissue of an organism [50]. Due attention, however, must be paid to the interrelation between different compartments of the organism and effects on the whole organism.

In in vivo experiments, silver was found to exhibit toxic properties that resulted in death, histopathological alterations in some organs, biochemical changes, and (rarely) effects on physiological functions. There was also evidence of increased ROS production, a reduction in the antioxidant defense capability, and oxidative stress [46, 47, 51].

In an incident described in [52], a pregnant woman was administered *in utero* 7 g of silver nitrate in the form of a 7% aqueous solution (a dose of ~64 mg of Ag per kg of body mass) and she died 3.5 h later with symptoms of acute circulatory failure. We note that in this case, where toxic silver nitrate was used, it is also important to take into consideration possible comorbidities, pregnancy, and, not unimportantly, the fact that the psychological state of the woman was laden with the necessity of abortion. Because these details were not reported, we cannot draw a more objective conclusion about the causes of her death.

Upon the acute exposure of *Danio rerio* fish to silver in the form of a salt (AgNO_3_) and 81-nm Ag NPs stabilized with polyvinylpyrrolidone (PVP), high mortality rates were observed upon an increase in the concentration of the potential toxin, along with stress signs such as more intensive swimming activity and attempts to escape the container [53]. Upon 24-h exposure to the ionic form of silver, LD_50_ was 28 μg/L. Upon 48-h exposure to Ag NPs, LD_50_ proved to be 84 μg/L. In addition, a higher amount of slime (presumably exuded from the gills) was observed at the bottom of the container after exposure to the NPs and ions. On the whole, the ionic form exhibited more pronounced toxic properties.

No noticeable hematologic and biochemical alterations were detected studying inhalation toxicity where rats were subjected for 28 days to Ag NPs with a size of 11–14 nm at concentrations of 1.73 × 10^4^, 1.27 × 10^5^, and 1.32 × 10^6^ cm^–3^ [54]. For the inhalation exposure of rats to NPs with a size of 12–15 nm over the same period of time, no histopathological changes in the mouth and lungs were observed [55]. In mice, however, short-term (14 days) intranasal exposure to Ag NPs with a size of 20 nm at a concentration of 1.91 × 10^7^ cm^–3^ caused changes in gene expression [56]. The subchronic (90 days) inhalation administration of Ag NPs with a size of 18 nm to rats resulted in slight dose-dependent inflammation of the lungs and changes in their function [57]. Furthermore, inhaled Ag NPs can make their way into the blood circulation system and reach extrapulmonary organs such as the liver and brain [54, 56].

The peroral administration of Ag NPs with a size of 40 nm at doses of 20 and 50 μg/day to BALB/C mice resulted in elevated levels of alanine aminotransferase and aspartate aminotransferase, as well as histological changes such as necrosis, hepatocyte inflammation, and associated lymphocyte aggregation in the liver tissue [59]. Similarly, the 60-day exposure of female Wistar rats to Ag NPs with a size of 10–40 nm at doses of 50 and 200 μg/day per animal caused a noticeable damage to mitochondria, an increase in serum creatinine levels, and the detection of markers of early toxicity such as KIM-1, clusterin, and osteopontin [60].

The effects of Ag NPs and silver salt (AgNO_3_) administered orally at equivalent doses to rats for 28 days were compared [61]. The study reported a more pronounced effect of Ag NPs, in comparison with silver nitrate, on blood biochemical markers: an increase in the erythrocyte and lymphocyte counts and a decrease in the thrombocyte count.

For long-term (4 and 6 months) daily oral exposure of C57BL/6 mice to PVP-capped Ag NPs with a size of 34 nm at a dose of 50 μg/day per animal, the CA2 hippocampus region was observed to become loose: neurons were distributed nonuniformly and sparse in comparison with the brain of the control animals [39].

For CBF1 mice, single enteral exposure to stabilized Ag NPs with a size of 30–60 nm at a dose of 4 mg/kg did not cause noticeable enterotoxic and hepatotoxic effects. Similarly, repeated exposure to these Ag NPs at doses below 0.45 mg/kg did not cause side effects. In an acute experiment, a higher NP dose caused increases in aminotransferases and urea as well as a shift in the albumin/globulin ratio, indicating the involvement of inflammatory processes. In addition, the relative mass of the liver of experimental animals was lower compared to the control. In a subacute experiment, the weight gain in groups that were administered Ag NPs at doses of 0.25–2.25 mg/kg was slower compared to the control, while in groups that received NPs at a dose of 2.25 mg/kg, the variation in the serum transaminase activity was significant, which was indicative of hepatosis. We note that the spleen and the liver of animals from groups in which the Ag NP doses were 0.45 and 2.25 mg/kg were more than twofold smaller compared to the control. Relatively small hyperemic regions and enlarged Peyer’s patches were observed in the intestines of some animals from groups exposed to Ag NPs at a dose of 2.25 mg/kg. Histologic study demonstrated the initial stages of inflammation of the liver and intestinal walls [48].

A dose-dependent anabolic effect was detected in mice subjected to the oral administration of sodium-citrate-stabilized Ag NPs for 30 days [2]. For a daily dose of 6.61 mg per 1 kg of body mass, the body weight gain remained proportional to the organ weight gain, which was indicative of the physiological character of the changes.

There are far fewer in vivo studies on the toxicity of silver in the salt form than in the form of NPs. Nonetheless, the observed tendency suggests that the ionic form exhibits more pronounced negative effects, which are more intense and develop faster. It is beyond argument that, in modeling the actual exposure of mammals to different forms of silver, it is important to highlight the factor of comorbidities such as hypertension, diabetes, and asthma, to name just a few [49]. The case study from clinical practice [52] described above reinforces this.

In some cases, the toxicity of silver is not considered to be a disadvantage at all. Thus, several modern studies reported of the antitumor effects of various compounds of the element under discussion [62, 63]. The possibility of using Ag NPs for the targeted delivery of antitumor and other types of drugs [64] is explored in connection with their affinity for specific cell organelles (mitochondria) [65] and apparently to certain body tissues, which will be considered below.

As a result, in studying the interaction between nanosilver and living systems, it becomes apparent that the substance from a medicine can be converted into a poison not only because of its dose but also due to the period of exposure of an organism to it. This fact suggests that a well-known dose measurement approach, in which the key parameter is the product of the dose and exposure period, can be applied to the assessment of risks associated with such substances.

## 4 BIOKINETICS OF SILVER

It is known that any substance that enters the body by various routes is involved in absorption, distribution, metabolism, and excretion (ADME) processes [66], each characterized by its own (normally dose-dependent) period. These periods form the biokinetic profile of the substance, which depends on a series of factors, including the route of entry.

When comparing the biodistribution of Ag NPs and silver salts, many researchers observed a common trend, and differences were only quantitative [61, 67]. Thus, for rats subjected to 28-day oral exposure to Ag NPs or equivalent doses of silver nitrate, the accumulation of this element was significantly higher in the case of salt [61], with the liver and the kidneys being major target organs. Lower amounts of silver were detected in the testicles and the spleen. The kinetics of accumulation of PVP-stabilized Ag NPs with a size of 14 ± 4 nm was compared with that of silver acetate [67]. The NPs and silver salt were characterized by similar bioaccumulation profiles. Silver was detected in the intestines, liver, kidneys, lungs, and brain. The silver salts, however, accumulated faster than the NPs, and the latter were actively excreted with faeces. Granules of silver and its compounds with S and Se were detected in the ileum of animals exposed to silver salts and NPs.

The accumulation kinetics of identical forms of silver and their affinity to specific organs vary considerably with the duration of exposure [68–71]. For single exposure to PVP-stabilized Ag NPs with a size of 34 nm, the levels of silver in the blood rose during the first hours after administration, and then it was transferred to the liver, kidney, and spleen [68]. In the case of repeated oral exposures to Ag NPs over one month, the levels of this element increased in the blood, liver, and brain. However, after exposure to Ag NPs was discontinued and distilled water was instead given to the mammals, the amount of silver excreted from the liver and blood during one month was around 85%, whereas only 5% was excreted from the brain. This phenomenon may be related to the presence of a considerable number of immune cells in the blood and the liver and diminished exocytosis in the brain [69]. The results described above were obtained by the highly sensitive technique of neutron-activation analysis. Using radioactive labeling, the silver content was measured in the organs of rats that were orally administered silver nitrate at a dose of 0.03 mg/L for one or two weeks [72]. Apparently, saturation levels were achieved fairly rapidly in different organs. Silver accumulated (in descending order) in the salens muscle, cerebellum, spleen, duodenum, and heart muscle. The accumulation of silver in organs and tissues that play an important part in motor functions may be critical in emergencies, when precision of movements is particularly important.

In a 28-day experiment in which rats were administered silver nitrate with a size of <15 nm and PVP-stabilized and unstabilized Ag NPs with a size of <20 nm [73], the resulting distribution profiles of silver in the body were similar. Silver accumulated (in descending order) in the liver, spleen, testicles, kidneys, lungs, and brain. The relative silver contents of the listed organs were higher in the case of exposure to the silver salt. At the same time, the presence of a stabilizing capping layer on Ag NPs had no effect on the biodistribution of silver. After exposure to silver was discontinued, the silver was efficiently excreted for 8 weeks from all of the organs except for the testicles and brain. The absorption of silver was found to occur in the intestine. In both cases, i.e., with Ag NPs and salts, silver accumulated in the form of NPs.

It was conclusively established that Ag NPs are able to pass through different physiological barriers in the body: blood-brain [68], blood-bile, blood-kidney, and blood-placental barrier, and others [18].

Consequently, silver displays a higher affinity for the liver, kidney, and spleen during the first days or even hours of exposure. However, as a result of its slow excretion, silver accumulates in the brain, lungs, and testicles and passes through blood-tissue barriers [74], as the time of exposure increases.

We note that all of the biokinetic experiments comparing the biodistribution of Ag NPs and silver salts yielded identical distribution profiles. The relative and absolute silver contents, however, were higher for exposures to silver salts than Ag NPs. The Ag NPs are eliminated from the body mainly with faeces.

## 5 EFFECT OF SILVER ON COGNITIVE FUNCTIONS IN MAMMALS

The effect of silver on the cognitive and behavioral functions in mammals should be considered toxic, i.e., neurotoxic, however, this phenomenon is addressed in a separate section of the present review.

It is known that in the Middle Ages attempts were made to treat psychiatric disorders, such as epilepsy, with silver nitrate as an anticonvulsant [75]. Nevertheless, known published sources highlight the negative facet of the effect that nanosilver has on the cognitive and behavioral functions in animals.

Thus, the effects that acute and subacute systemic intravenous administrations of Ag NPs with a size of ~25 nm had on the memory, learning abilities, social behavior, and motor functions of BALB/C mice were studied [76]. Worsening of all of the parameters listed above were observed.

In Wistar rats, subacute oral exposure to BSA-stabilized (bovine serum albumin) Ag NPs with a size of 20 nm during a 28-day period resulted in negative effects such as worsening memory and brain plasticity [77], with silver accumulating in the ionized form in the brain.

Behavioral changes and alterations of long-term contextual memory were observed in C57Bl/6 mice subjected to oral exposure to PPV-stabilized Ag NPs with a size of 34 nm [17]. In doing so, the cognitive functions underwent three different stages: an increase in anxiety, the involvement of adaptation mechanisms, and the dysfunction of long-term contextual memory. Earlier, such NPs were shown to be able to pass through the blood-brain barrier [68].

Cognitive and behavioral dysfunction may be a direct consequence of the accumulation of silver in the brain and its structures [39, 77, 78]; however, an overlap with the overall effect on the organism cannot be ruled out.

The first hypothesis is supported by studies demonstrating a lack of the negative effect of Ag NPs on the gut microbiome, which potentially can be the main target for such NPs [79, 80]. Nevertheless, the suppressive action on several transitory components represented by conditionally pathogenic species of microorganisms was observed [80], along with the considerable growth of lactic-acid bacteria [79]. Available publications lack information on the effect that the ionic form of silver has on the gut microflora.

It can be of interest to study the effect of silver in the salt form on the cognitive functions, but such works were not identified.

## 6 ARGYROSIS

It is known from clinical practice that the exposure of living organisms to silver may cause a disease known as argyrosis. The condition clinically presents as a grayish-brown or black-brown coloration of the skin, mucosa, tissues of internal organs and eyes resulting from the deposition of silver in them [81]. Localized and generalized argyrosis are distinguished [82]. Localized argyrosis consists in local changes in the coloration of the skin surface and mucosa, which typically occur at the site of contact with silver items. Generalized argyrosis develops as a result of long-term exposure to silver and affects different organs and body systems.

Occupational exposure, medical and paramedical practices, and the use of cosmetics were identified among the causes of argyrosis [83]. Argyrosis may develop as a result of exposure to metalic silver or its soluble forms. For instance, argyrosis of the fingers [84] and fingers and hands [85] was diagnosed in silversmiths. Localized argyrosis of the skin [86] and eyes [87] was observed in jewellers. A young lady developed epidermal necrolysis affecting nearly 100% of the skin surface. She was prescribed dressings containing Ag NPs for an indefinite time, and four years later she was diagnosed with localized argyrosis [88].

Generalized argyrosis was observed in a female patient who had mouth ulcers and applied 10% silver nitrate to the tongue for a year [89]. Gray-blue diffuse coloration of the skin surface was observed in a 59-year-old male with chronic laryngitis who was self-medicating with Ag-containing pharmaceuticals in spray form over a 15-year period. A patient died of small-cell anaplastic lung cancer, and the autopsy showed coloration of the renal cortex and vascular plexuses. Black granules containing silver were found in all of the studied organs except for the brain parenchyma [90].

A 46-year-old woman developed pronounced pigmentation as a result of her using silver nitrate on bleeding gums 3 times a week for 26 months [91]. Liver biopsy revealed the presence of silvery-colored areas around portal regions and the central vein. No significant reduction in skin pigmentation was observed in the following two years. In subsequent operations on the abdomen, pancreas, stomach, liver capsule, spleen, intestines, and peritoneum, changes in the coloration of these organs and the skin were observed. The pancreas was affected by pigmentation to the largest extent and had a silvery appearance. Stomach biopsy showed the deposition of silver granules in the connective tissue.

A great number of similar cases is known. Generalized argyrosis typically develops as a result of long-term administration of Ag-containing solutions, and the localized form develops due to contact with metallic silver or nanosilver.

Overall, argyrosis is not a life-threatening condition and is considered to be a cosmetic issue.

## CONCLUSIONS

As a result of the increased circulation of different forms of silver in the food and cosmetic industries, pharmacology, and medicine, its levels in the ecosphere and biosphere have risen, and exposure of the human organism to this element has increased. This highlights the importance of our understanding of mechanisms of interaction between silver and living objects, which begin with cellular processes and cumulate in developing a risk assessment for organisms overall. It is already clear that these aspects contain more questions than answers. Admittedly, silver in soluble forms may pose certain risks regarding its toxic and neurotoxic effects on organisms and especially due to its possible recycling in the environment.

Nevertheless, we should not overestimate the risks associated with the uses of silver. At present, in clinical practice, the only condition reliably associated with exposure to silver as argyrosis is known. The negative (toxic and neurotoxic) effects of silver may be caused by a combination of factors, including psychological aspects, and typically they cannot be predicted reliably.

The importance of stringent control over the content of silver in various ecological niches and areas of the technosphere is obvious. Increased release of this element into the environment may contribute to alterations of established biochemical equilibrium and cause new hard-to-foresee diseases.

Soluble forms of silver (as ions (salts)) produce more powerful and rapid toxic effects, and this characterizes the accumulation of silver as well. In this respect, nanosilver comes second, especially when silver particles are capped with stabilizing layers, which increases their solubility. Bulk silver is the most benign. This is likely to be related to the fact that it is the ionized form of silver that more actively interacts with living objects, which also agrees with the high inertness of metallic silver.

The growing use of soluble forms of silver, particularly as colloidal NPs, increases associated risks, because these forms are more bioavailable and produce lasting effects. As the duration of exposure to nanosilver increases, a need arises to revise the current strategies for assessing its toxicity to living organisms. This opens up the possibility for the dose-measurement approach which incorporates both the intensity and period of exposure and corresponds to the effect of Ag-NP accumulation in a range of organs and tissues.
